# Angiogenesis and Lymphangiogenesis in the Spectrum of Leprosy and Its Reactional Forms

**DOI:** 10.1371/journal.pone.0074651

**Published:** 2013-09-06

**Authors:** Cleverson Teixeira Soares, Patrícia Sammarco Rosa, Ana Paula Fávaro Trombone, Luciana Raquel Vicenzi Fachin, Cássio César Ghidella, Somei Ura, Jaison Antonio Barreto, Andréa de Faria Fernandes Belone

**Affiliations:** 1 Laboratory of Anatomic Pathology, Lauro de Souza Lima Institute, Bauru, São Paulo, Brazil; 2 Division of Research and Education, Lauro de Souza Lima Institute, Bauru, São Paulo, Brazil; 3 Ambulatory of Leprosy, Jardim Guanabara Health Center, Rondonópolis, Mato Grosso, Brazil; 4 Ambulatory of Leprosy, Division of Dermatology, Lauro de Souza Lima Institute, Bauru, São Paulo, Brazil; Medical University Innsbruck, Austria

## Abstract

**Background:**

Angiogenesis and lymphangiogenesis are the processes of neovascularization that evolve from preexisting blood and lymphatic vessels. There are few studies on angiogenesis and none on lymphangiogenesis in leprosy. Thus, the role of neovascularization in the pathophysiological mechanisms of the disease was studied across the spectrum of leprosy, its reactional states and its residual lesions.

**Methodology/Principal Findings:**

Seventy-six biopsies of leprosy skin lesions and seven healthy controls were selected. Fifty-five serum samples were used for the detection of CD105 by ELISA. Histological sections were stained with antibodies against CD31 (blood and lymphatic vessels), D2-40/podoplanin (lymphatic vessels), and CD105/endoglin (neovessels). Microvessels were counted in 100 high-power fields (400x) and the number of vessels was evaluated in relation to the extension of the inflammatory infiltrate (0-3), to the bacillary index (0-6) and to the clinical forms. Angiogenesis, as marked by CD31 and CD105, was observed across the leprosy spectrum, compared with the controls. Additionally, there was a positive correlation between these markers with extension of the infiltrate (p <0.0001). For D2/40, lymphangiogenesis was observed in the tuberculoid form (p <0.0001). There was no statistical significance for values of CD105 detected in plasma by ELISA.

**Conclusions/Significance:**

Angiogenesis is present across the spectrum of leprosy and in its reactional forms. The increase in the number of vessels, as detected by CD31 and CD105 staining, is related to the extension of the inflammatory infiltrate. Samples from reactional lesions have a higher number of CD31+ and CD105+ stained vessels, which indicates their involvement in the pathophysiological mechanisms of the reactional states. The regression of lesions is accompanied by the regression of neovascularization. Drugs inhibiting angiogenesis may be relevant in the treatment of leprosy, in addition to multidrugtherapy, and in the prevention of the development of reactions.

## Introduction

Leprosy is a chronic infectious disease caused by *Mycobacterium leprae*. The bacillus has tropism for the peripheral nervous system, making leprosy a predominantly neural disease. The classification of Ridley and Jopling (R&J) divides leprosy into two polar forms: tuberculoid (TT) and lepromatous (LL). An immunologically unstable intermediate group separates the two polar forms and is divided into three subgroups: borderline tuberculoid (BT), borderline-borderline (BB) and borderline-lepromatous (BL). These groups are classified according to clinical characteristics, skin smear bacillary index and histopathological features [1]. Reactions are the abrupt onset of cutaneo-neural lesions, which are sometimes intensive and destructive. These may occur at variable times during leprosy progression. There are two types of reactions in leprosy. One occurs in patients in whom specific cellular immunity against *M. leprae* is preserved to some degree; this reaction is called a type "1" reaction or Reversal Reaction (RR). The second type of reaction occurs in patients in whom cellular immunity is only slightly preserved or is virtually absent and is called a type "2" reaction or erythema nodosum leprosum (ENL). Leprosy reactions are important events in the evolution of leprosy, and currently no specific treatment can prevent the occurrence of these phenomena. In general, during reactional episodes, it is not uncommon to observe worsening of the neurological injuries, which can cause permanent functional disabilities [2].

Angiogenesis and lymphangiogenesis are the processes of neovascularization from pre-existing blood and lymphatic vessels. These phenomena are regulated by endothelial growth factors and their receptors and can be observed both in physiological and pathological processes [3]. There are many studies on angiogenesis and lymphangiogenesis in neoplasias, and recently, the importance of angiogenesis has been recognized in inflammatory and infectious processes [4]. Currently, new angiogenesis inhibitors, which can normalize or block angiogenesis, are being developed for the treatment of inflammatory diseases and neoplasias [5].

Only a few studies have been conducted on angiogenesis in leprosy, and none have been conducted on lymphangiogenesis. Some studies have correlated the neovascularization observed in leprosy with increased bacillary index and disease progression [6,7]. However, the occurrence of neovascularization in reactional episodes or in regressive leprosy lesions has not been reported. Therefore, more detailed studies on angiogenesis and lymphangiogenesis in leprosy are important for the understanding of its pathophysiological mechanisms and to identify new therapeutic targets that may aid the treatment of the disease.

The aim of this study was to evaluate angiogenesis and lymphangiogenesis across the spectrum of leprosy, in its reactional episodes and in its residual lesions by immunohistochemistry (IHC), using the markers CD31, CD105/endoglin and D2-40/podoplanin [8–11]. Additionally, 55 samples were used for the detection of CD105 in the serum by ELISA. CD105/endoglin is a co-receptor for the transforming growth factor-β (TGF-β1) molecule, a multifunctional cytokine that is involved in many physiological and pathological processes and plays a central role in angiogenesis [11,12]. CD105 is mainly expressed in the endothelium of neovessels. Several studies have suggested that CD105/endoglin is a specific marker of neovascularization in several cancer processes. It is strongly expressed in neovessels and is usually absent in normal vessels. In the skin, it is expressed almost exclusively in neovessels, and it is not present in the common vessels and other components of the skin. Some studies with neoplasias show contradictory results in respect to anti-CD105 levels in the serum by ELISA [13], however, in leprosy, CD105 might be a marker of severity of the disease, as well as a predictive marker for reactional episodes. CD31, also called endothelial platelet adhesion glycoprotein-1 molecule, is a 130-kDa transmembrane protein that is expressed on monocytes, platelets, "T" lymphocytes subsets and in blood and lymphatic vessels. CD31 shows strong positive expression in the blood and lymphatic vessels of the skin, while all other skin components are negative for CD31 expression [9]. D2-40/podoplanin is a monoclonal antibody of subclass IgG2a; it is directed against the M2A oncofetal antigen that is usually expressed in germ cell tumors and fetal testis tissue [10]. In addition, it is recognized as one of the most sensitive and specific markers that can be used to differentiate lymphatic and blood vessels.

## Materials and Methods

### Ethical Statement

This is a retrospective observational study that used paraffin blocks from archive material of previously diagnosed leprosy cases, obtained from the Department of Pathology of Lauro de Souza Lima Institute (ILSL). The study was approved by the Research Ethics Committee of the ILSL. All samples were anonymized. Written consent was obtained from participants who accepted to have material from healthy skin collected to compose the control group.

### Selection of Cases

**Figure 1:**
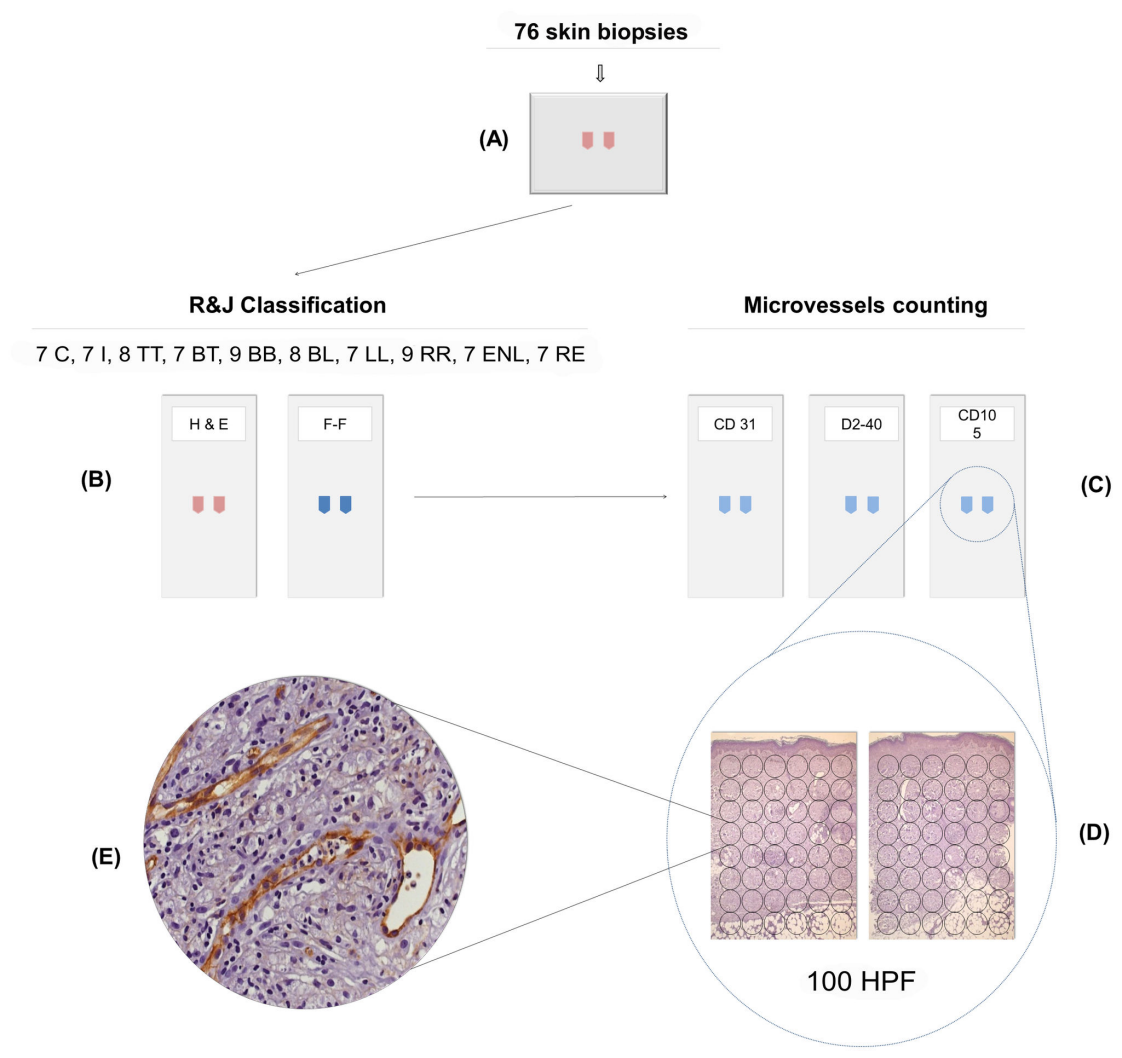
Experimental design. (A) Seventy-six paraffin blocks were selected. The samples constituted punch biopsies of patients in the leprosy spectrum, reactional cases, residual leprosy and controls (n=76). (B) The sections (4µm) were stained by HE and Fite-Faraco (R&J histopathological classification) and (C) immunohistochemistry using antibodies against CD31, D2-40 and CD105 for the counting of microvessels. (D) For each marker, the microvessels were counted in 100 hpf, within the entire thickness of the dermis and part of the subcutaneous tissue. (E) A field immunostained for CD105 in ENL lesion. (*) 46 patients, six TT, six BT, six BB, seven BL, seven LL, seven RR and seven ENL, also had blood samples taken. Nine healthy controls (C) were included in the ELISA test for serum detection of CD105.

Seventy-six blocks of surgical specimens from skin biopsies (punch 5 or 6) that were taken from the skin lesions of leprosy patients and controls between January of 2007 and June of 2011 were selected for retrospective evaluation. Biopsies of newly diagnosed patients were allocated to the following subgroups: seven indeterminate (I), eight tuberculoid (TT), seven borderline tuberculoid (BT), nine borderline-borderline (BB), eight borderline-lepromatous (BV), seven lepromatous (LL). Biopsies from reactional patients were collected either at diagnosis, or during or after multidrugtherapy (MDT) treatment, however, none of them had received corticosteroids or thalidomide to treat the reactional episodes. The reactional patients were then allocated to other sub-groups: nine type "1" reaction (RR) and seven type "2" reaction (ENL). The control was composed by seven biopsies from healthy individuals (C) collected from upper and lower limbs and trunk. These biopsies were collected from individuals that came to the ambulatory of the institute, with suspicion of a dermatological disorder, however, the clinical and histopathological evaluation resulted negative for infectious, inflammatory, metabolic, neoplastic or genetic disease, and also, the referee patients was not taking any drugs routinely. Additionally, a sub-group of seven residual lepromatous leprosy lesions (RE), composed by samples of regressive lesions taken more than ten years after the end of specific MDT treatment and who presented with clinical and histopathological features of residual lesions was included (Figure 1). The inclusion criteria used were as follows: 1) samples without ulceration of the skin, 2) samples with representation from all layers of the skin and subcutaneous tissue, and 3) samples that were not taken from scalp, face, soles and palms, that could show variable number of vessels, compared to other skin parts.

Forty-six of the 76 selected samples also had paired serum samples collected at the same time of the skin biopsy, and these cases comprised six TT, six BT, six BB, seven BL, seven LL, seven RR and seven ENL.A group of nine sera from healthy individuals were retrieved from the sera bank of the Institute to be used as controls for the anti-CD105 serological assay.

### Histological Analysis

New histological hematoxylin-eosin (HE) and Fite-Faraco sections were prepared for all selected blocks. One pathologist (CTS) evaluated the slides for classification according to the R&J criteria, extension of the inflammatory infiltrate, bacillary index and counting of microvessels. With respect to the inflammatory infiltrate, the histological sections were scanned at low magnification (40x) for the semi-quantitative definition of infiltrate score, defined as absent or minimum (zero) when infiltrates constituted up to 5% of the extension of the histological sections, mild (1+) when infiltrates constituted between 5 and 25%, moderate (2+) when infiltrates constituted between 25 and 50% and intense (3+) when infiltrates constituted more than 50% of the histological section. The bacillary index (BI) ranged from zero to 6+, as defined in the literature [1].

### Immunochemical Technique (IHC)

The IHC reaction was performed according to standard protocols, using the indirect streptavidin-biotin peroxidase method for CD31 (LSAB - Dako, California / USA) and envision for CD105 and D2-40 (Dako, California / USA) in accordance with the manufacturer’s recommendations. The colorimetric revelation was performed using 3'-3'-diaminobenzidine (DAB) with hydrogen peroxide as the substrate (Dako, California / USA). Slides were counterstained with hematoxylin (Merck KGaA, Darmstadt, Germany). Negative and positive control biopsy materials, both internal and external, were also evaluated. The primary antibodies used were monoclonal "mouse anti-human” and are listed as follows: (a) CD31 endothelial cell (clone JC/70A, 1:100, Dako, Glostrup, Denmark), (b) D2-40/podoplanin (cloneD2-40, 1:200, Dako, Glostrup, Denmark), and (c) CD105/endogolin (clone4G11, 1:100, Novocastra, Newcastle, UK). The histological sections were scanned at high magnification (400x) for the counting of microvessels without prior knowledge of the classification of the samples. A total of 100 fields were sequentially evaluated for vessel number, starting at the papillary dermis so that the entire thickness of the dermis and part of the subcutaneous tissue were examined (Figure 1). The fields were counted in two histological sections, each corresponding to a respective half of the specimen (punch biopsy). The slides were analyzed under a Nikon Eclipse 50*i* light microscope (Nikon Corporation, Japan), in which each high power field (hpf) corresponded to 0.152 mm^2^. The sum of vessels per 100 hpf was calculated. Individual capillaries were counted in each section, and arterioles and venules were excluded. Any intensity of immunostained vessel was considered to be positive. The values obtained for the three markers were evaluated after counting the vessels in 100 hpf. Statistical analysis of the following groups was carried out: (a) forms across the leprosy spectrum, reactional states, residual leprosy and control, (b) bacillary index, (c) extension of the inflammatory infiltrate, (d) RR versus forms in the spectrum prone to developing RR (TT + BT + BB + BL), (e) ENL versus forms in the spectrum prone to developing ENL (BL + LL), and (f) RR versus ENL.

### ELISA for Serological Detection of CD105

The Quantikine ELISA kit - Human endoglin/CD105 was used for the serological detection of CD105 in the 55 samples. The assay was performed according to the manufacturer’s instructions (R & D Systems, Minneapolis, USA).

### Statistical Analysis

The data were represented as the mean ± SEM and analyzed using GraphPad Prism version 5.0 for Windows (GraphPad Software, San Diego, CA). Statistical significance among groups was calculated by the Kruskal-Wallis test, followed by Dunn’s Multiple Comparison test. Values of p<0.05 were considered as significant.

## Results

**Figure 2:**
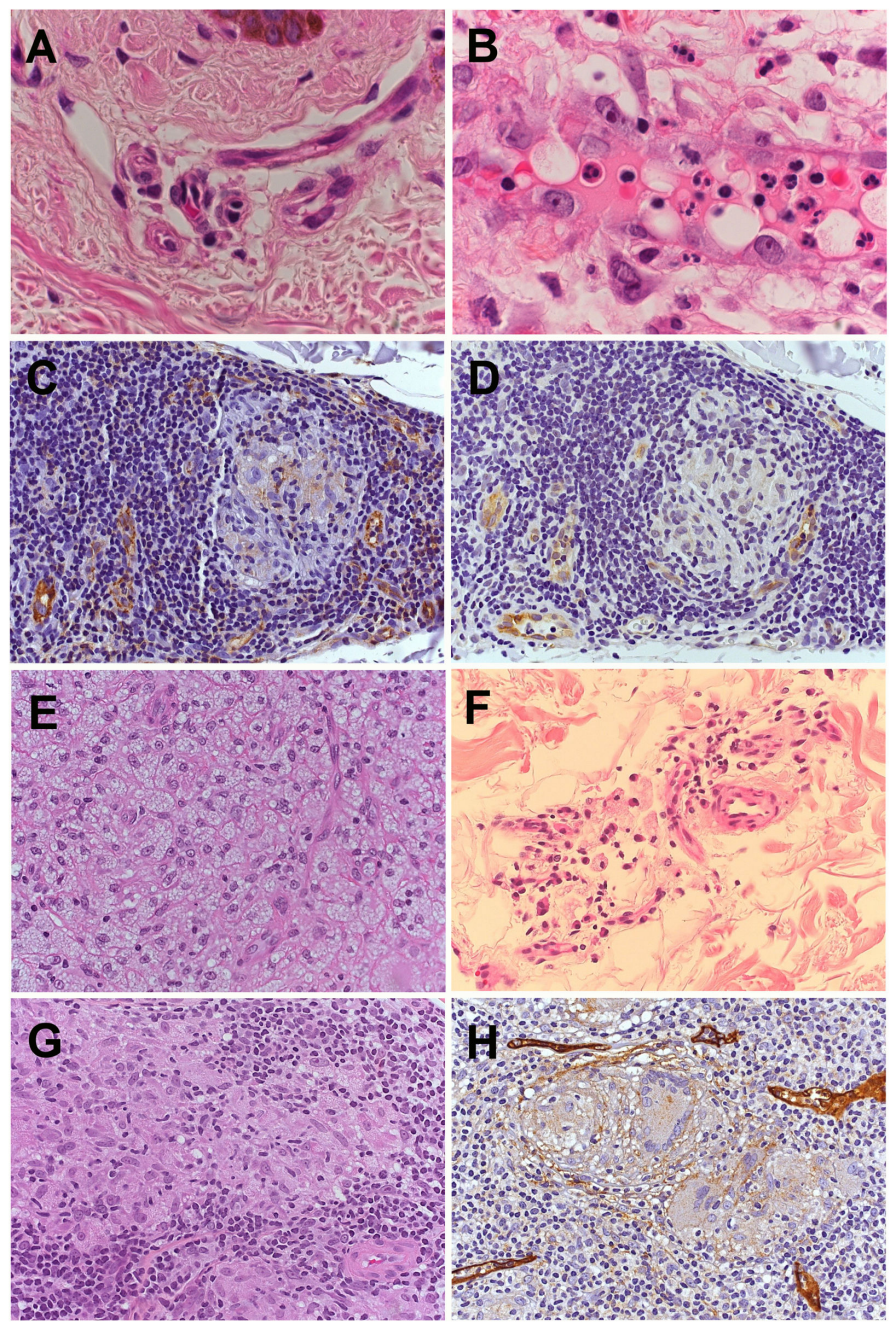
Histopathological pictures. (A) A normal capillary vessel in the papillary dermis, comprising endothelial cells with fusiform shape and showing elongated nuclei (HE, 1000x). (B) The endothelium of an ENL skin lesion, comprising endothelial cells with large and vacuolated cytoplasm, large nuclei, one or more nucleoli and mitoses. The lumen of the capillaries and the interstitium are filled by neutrophils, macrophages, lymphocytes and plasma cells, among other cells (HE-1000x). (C) Capillaries positive for CD31 inside and surrounding the tuberculoid granuloma (IHC, CD31- 200x). (D) Capillaries positive for CD105 inside and surrounding the tuberculoid granuloma (IHC, CD105- 200x). (E) A lepromatous lesion showing multivacuolated histiocytes permeated by capillary vessels (HE- 400x). (F) A residual lesion showing regressive granulomas with a few histiocytes with multivacuolated cytoplasm (HE- 400x). (G) A tuberculoid leprosy case showing a granuloma comprised of epithelioid macrophages in the center and lymphocytes in the periphery (HE- 400x). (H) Lymphatic vessels around the tuberculoid granuloma containing multinucleated giant cells (IHC, D2-40, 200x).

Among the 76 selected samples, 55 were males and 21 were females. The male predominance was observed in all clinical forms of the leprosy spectrum and also in the reactional cases. The mean age was 44 years and ranged from 8 to 88 years. Hematoxylin-eosin (HE) histological sections of leprosy lesions showed distinct histological changes in the blood capillaries compared with control sections. In general, the vessels in C, I, and RE exhibited fusiform endothelium with elongated nuclei and without nucleoli (Figure 2a). In the leprosy spectrum, the endothelial cells were larger, with round nuclei and one or two small nucleoli, which are evidences of endothelial cells in proliferation. In the reactional cases, especially in ENL samples, the endothelial cells were large, sometimes epithelioid, with multivacuolated cytoplasm and prominent nucleoli, and showed occasional mitoses (Figure 2b).

With respect to the number of microvessels evaluated in 100hpf, significant differences were observed between several of the groups when compared with the clinical form, reactional episodes and control (Table 1). In respect to CD105 positivity, it was significantly higher in the polar lepromatous group (LL) and reactional groups (RR and ENL) when compared with groups I, RE and C (Table 1). Additionally, there was significantly higher CD105 staining in the BL group compared with group C.

**Table 1:** Mean number of vessels for all leprosy groups and control (n=76).

Groups	CD31	D2-40	CD105
C (n=7)	705 (619-849)**^a^**	97 (75-114)	**0**(0)**^f^**
I (n=7)	781 (578-925)**^b^**	101 (79-134)	**2**(0-5)**^g^**
TT (n=8)	1101 (961-1305)	187 (124-261)**^e^**	115 (26-338)
BT (n=7)	838 (728-1006)	133 (80-201)	139 (58-293)
BB (n=9)	774 (642-911)**^c^**	89 (60-116)	65 (14-177)**^h^**
BL (n=8)	837 (643-942)	97 (74-117)	193 (35-343)
LL (n=7)	1124 (671-1572)	117 (65-185)	436 (155-1113)
RR (n=9)	989 (802-1183)	148 (75-290)	337 (196-520)
ENL (n=7)	1501 (1132-2156)	104 (85-119)	890 (572-1245)
RE (n=7)	758 (611-908)**^d^**	111 (84-160)	**6**(0-15)**^i^**

Mean values (minimum - maximum) of the number of vessels counted using three markers (CD 31, D2-40 and CD 105) for all clinical forms of the leprosy spectrum, reactional cases, residual leprosy and control group. P<0.05: (CD31) ^a^ C versus TT, LL and ENL; ^b^ I versus ENL; ^c^ BB versus TT and ENL; ^d^ RE versus TT and ENL (D2-40). ^e^ TT versus BB, BL and C (CD105). ^f^ C versus BL, LL, RR and ENL; ^g^ I versus LL, RR and ENL; ^h^ BB versus ENL; ^i^ RE versus LL, RR and ENL.

When the bacillary index was evaluated with respect to the positive staining of the different markers, a significant difference was only observed for CD105. CD105 expression was significantly higher in patients with scores of 3 and 6 when compared with those with scores of 0 and 1 (Table 2). With respect to the extension of the infiltrate, significantly higher positive CD31 staining was observed in patients with scores of 2 or 3 when compared with those with scores of 0 or 1 (Table 3). For the CD105, there was significantly higher number of positive stained vessels in patients with scores of 1, 2 or 3 compared with patients with a score of 0, and in patients with scores of 2 or 3 when compared with patients with a score of 1 (Table 3). There was no significant difference in D2-40 expression between the patients with different scores.

**Table 2:** Mean number of microvessels stratified by bacillary index (BI) for leprosy groups (n=69).

Bacillary index	CD31	D2-40	CD105
0**(n=13)**	922 (611-1294)	151 (84-290)	**87** (0-455)**^a^**
1**(n=13)**	878 (578-1305)	138 (82-261)	**80** (0-437)**^b^**
2**(n=8)**	843 (811-1006)	126 (80-201)	182 (58-347)
3**(n=6)**	1335 (794-2156)	110 (85-160)	685 (43-1245)
4**(n=6)**	833 (642-1144)	106 (87-140)	134 (14-403)
5**(n=10)**	966 (643-1652)	92 (60-119)	285 (28-984)
6**(n=13)**	1033 (671-1572)	107 (79-185)	386 (165-1113)

Mean values (minimum - maximum) of the number of microvessels counted using three markers (CD30, D2-40 and CD105), and stratified by BI for all clinical forms of the leprosy spectrum, reactional states and residual leprosy groups. P<0.05: (CD105) ^a^ 0 versus 3 and 6; ^b^ 1 versus 3 and 6.

IB “0” (n=13): four TT, two RR and seven RE.

IB “1” (n=13): seven I, four TT, one BT and one RR.

IB “2” (n= 8): six BT and two RR.

IB “3” (n=6): one BB, one RR and four ENL.

IB “4” (n=6): four BB and two RR.

IB “5” (n=10): four BB, three BL, one RR and two ENL.

IB “6” (n=13): five BL, seven LL and one ENL.

**Table 3:** Mean number of vessels compared to extension of the inflammatory infiltrates in leprosy groups (n=69).

Inflammatory infiltrate	CD31	D2-40	CD105
0**(n=14)**	770 (578-925)**^a^**	106 (79-160)	**4**(0-15)**^c^**
1**(n=23)**	823 (642-1098)**^b^**	110 (80-180)	101 (14-239)**^d^**
2**(n=23)**	1041 (792-1305)	140 (60-290)	308 (32-714)
3**(n=9)**	1417 (763-2156)	105 (65-185)	767 (301-1245)

Mean values (minimum - maximum) of the number of vessels counted using three markers (CD 30, D2-40 and CD105) in relation to the extension of the inflammatory infiltrates (scores), in all clinical forms of the leprosy spectrum, reactional states and residual leprosy groups. Scores: “0” from 0 to 5%; “1” from 6% to 25%; “2” from 26% to 50%; “3” more than 50%. P<0.05: (CD31) ^a^ 0 versus 2 and 3; ^b^ 1 versus 2 and 3; (CD105) ^c^ 0 versus 1, 2 and 3; ^d^ 1 versus 2 and 3.

Score “0” (n= 14): seven I and seven residual.

Score “1” (n= 23): four TT, six BT, seven BB, three BL, one LL and two RR.

Score “2” (n= 23): four TT, one BT, two BB, four BL, two LL, seven RR and three ENL.

Score “3” (n= 9): one BL, four LL and four ENL.

**Figure 3:**
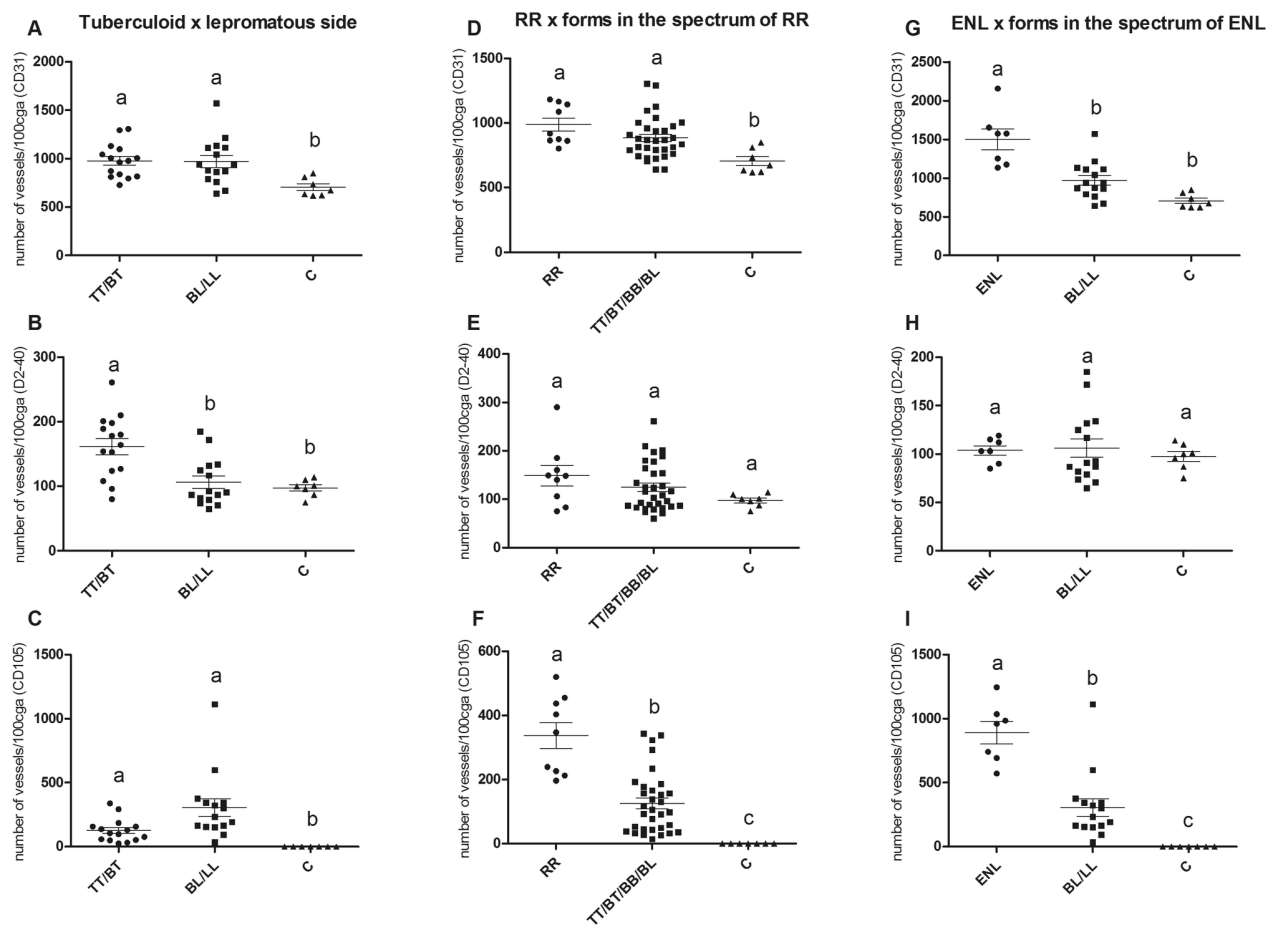
Microvessels counting. Evaluation of positive staining for the three markers (CD31, D2-40 and CD105) in comparison with polar forms of the leprosy spectrum, reactional groups (RR and ENL) and the clinical forms of the reactional groups (A-I). Different letters indicate statistical significance (P<0,05), equal letters indicate no significance.

When the opposite extreme sides of the leprosy spectrum, tuberculoid (TT/BT) and lepromatous (BL/LL), were compared to controls, CD31 and CD105 positive stained vessels were significantly higher in patients with both tuberculoid and lepromatous sides compared with controls (Figure 3A and 3C). Patients on the tuberculoid side of the disease had a significantly higher number of vessels that stained positively for D2-40 compared with the control patients and patients on the lepromatous side of the disease (Figure 3B). The analysis of the reactional groups, RR and ENL, and their respective clinical forms, TT/BT/BB/BL for RR and BL/LL for ENL, showed significant differences in CD105 staining among all groups evaluated, with the highest number of positively stained vessels observed in the reactional group, followed by the clinical forms and the control group (Figure 3F and 3I). For D2-40, no significant difference in expression was observed between the groups evaluated (Figure 3E and 3H). There was a significantly higher number of vessels positive for CD31 staining in the RR and clinical forms compared with the control group (Figure 3D). In addition, there was a significantly higher difference in the number of vessels positive for CD31 in the ENL group compared with the clinical forms and control group (Figure 3G).

**Figure 4:**
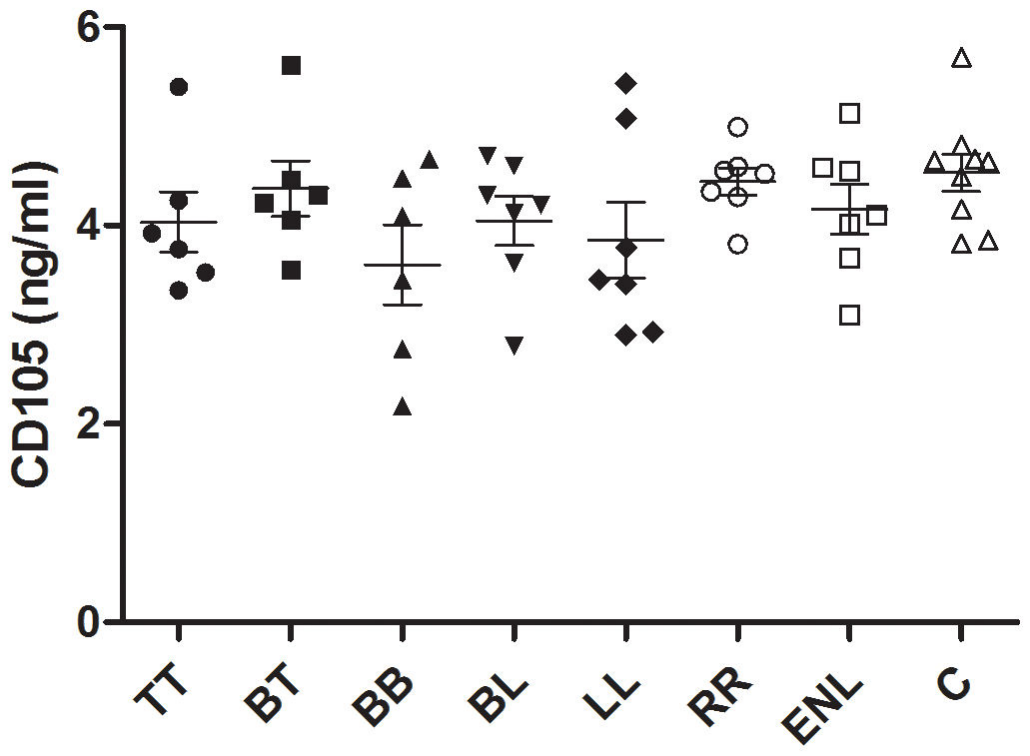
Anti-CD105 serum levels detected by ELISA. Detection of antibodies against CD105 in sera of leprosy patients across the spectrum, in reactional groups and controls. Data reported as means ± SD absorbance. Results show that absorbance was not significantly different between the evaluated groups.

The CD105 values detected in the serum by ELISA are detailed in Figure 4. There was no statistically significant difference between groups in the spectrum, reactional states, residual leprosy lesions and control.

## Discussion

Angiogenesis is the process of the formation of new vessels from preexisting vessels, and it has important roles in physiological processes such as embryogenesis as well as in pathological processes. In pathological processes, angiogenesis generates abnormal vessels, as the newly formed vessels are often irregular, unevenly distributed and show heterogeneous structure and function, with the formation of arteriovenous shunts and variable fenestrations and showing hyperpermeability to plasma proteins and cells. Angiogenesis is involved in several biological processes, both physiological and pathological, such as cell proliferation, migration, cell differentiation and interaction [14].

Angiogenesis during neoplastic processes has received great attention in recent decades. The neovascularization induced by cancer cells is necessary to support the proliferation of cancer cells, their invasion into adjacent tissues and metastasis [15]. Recently, more importance has been given to studying angiogenesis in non-neoplastic diseases, such as inflammatory, autoimmune and infectious diseases. Studies have shown roles for angiogenesis in Crohn’s disease, Rheumatoid Arthritis, Psoriasis and Rosacea [16,17]. In chronic inflammatory processes, there are close relationships between angiogenesis and the cells that constitute the inflammatory infiltrate. Cytokines and pro-inflammatory chemokines, secreted during inflammatory processes, are potent activators of endothelial cells, inducing neovessels formation and proliferation as well as vasodilation, fluid leakage and the recruitment and adhesion of inflammatory cells to endothelial cells [17,18].

Only a few studies in the literature have addressed angiogenesis in leprosy, and none of them compared the clinical forms to the reactional states and the residual lesions. Antunes et al. found two distinct architectural patterns while investigating the microvasculature in leprosy skin lesions by immunohistochemistry using anti-factor VIII and *Ulex* *Europaeus* -Lecithin-1 as markers. In lepromatous lesions, tortuous vessels were noted between macrophages containing bacilli. In tuberculoid lesions, the microvessels were observed only at the periphery but not within the granulomas [6]. In the present study, CD31+ microvessels were identified in lesions from the tuberculoid side of the spectrum, including TT and BT, both in the periphery and within the granulomas, with the predominance in the periphery. CD105+ vessels were also detected within and outside of the granulomas, indicating that they were neovessels that had been induced to support the granulomas (Figure 2c and 2d).

Bhandarkar et al. evaluated microvessels by IHC with anti-CD31 and found that the average number of microvessels in BT, BB and LL forms was significantly higher than in indeterminate lesions and concluded that the increased bacterial load was associated with increased angiogenesis [7]. In the present study, the number of microvessels evaluated by anti-CD31 was significantly increased (P <0.05) in most forms of the leprosy spectrum and in reactional states, with the exception of indeterminate, BB and residual lesions, compared with the control. However, there was no direct relationship between the number of microvessels and the progression of leprosy across the spectrum or with the BI. In contrast, a correlation between the extension of the inflammatory infiltrate and the number of microvessels was observed (Tables 1, 2 and 3). The discrepant results observed are most likely due to the different evaluation methods used in the two studies. Bhandarkar et al. analyzed 32 samples, four I, five TT, 12 BT, three BB, three BL and five LL, and the enumeration of microvessels was performed in hot spots, which are areas determined by the observers to represent the fields of greatest vascular density within a given section [7,19]. In the present study, besides the leprosy spectrum, control, reactional (RR and ENL) and residual samples were evaluated. Additionally, microvessels were counted in 100 hpf with high power objective (400x), and the entire thickness of the dermis and the subcutaneous tissue was evaluated (Figure 1).

Significantly higher values of CD31+ and CD105+ microvessels were observed with respect to the extension of the inflammatory infiltrate, followed by the clinical forms of the spectrum and then the BI (Tables 1, 2 and 3). These results indicate a positive correlation between the extension of inflammatory infiltrate and the number of microvessels (Figure 3) and suggest that granulomas induce angiogenesis to provide the necessary support for their maintenance and expansion. The mechanisms regulating this process are not yet fully elucidated. It is noteworthy that despite the larger number of vessels in lesions on the tuberculoid side (TT/BT) than in BB and BL lesions, angiogenesis in these latter forms is likely greater because these lesions are more numerous and the patients present systemic involvement, which is in contrast to the few skin lesions found in patients on the tuberculoid side of the spectrum.

Because angiogenesis was observed across the leprosy spectrum and in reactional states, patients, especially those showing larger number of vessels, such as those of the subtypes LL, RR and ENL, could benefit from treatment with anti-angiogenic drugs. There are some literature reports that support this hypothesis. El-Khalawany et al. described that the addition of minocycline, a drug with anti-angiogenic action, to the conventional treatment of leprosy decreased BI and the onset of reaction episodes when compared with conventional treatment alone [20,21]. Additionally, thalidomide and similar drugs used in ENL treatment have antiangiogenesis as their major anti-inflammatory mechanisms [22,23].

When comparing the number of vessels in reactional cases and the forms within the leprosy spectrum [RR x (TT + BT + BB + BL) and ENL x (BL + LL)], higher numbers of vessels were observed in reactional compared with non-reactional forms (Table 1, Figure 3). As reactions are generally abrupt processes, it is likely that some of these vessels detected by both CD31 and CD105 also develop abruptly as a result of the reactional episode. Angiogenesis occurs abruptly in reactive lesions, for example, in pyogenic granuloma, indicating that neovascularization plays an important role in the triggering and maintenance of reactional episodes [24]. Therefore, blocking vascular proliferation with anti-angiogenic drugs could be an important factor for the treatment of reactions and for preventing reactions or decreasing their intensity. When comparing the reactional states (RR x ENL), we observed that the number of new vessels was significantly higher in ENL than in RR, indicating that anti-angiogenic therapy might have greater benefits in patients with ENL (Figure 3).

The data also shows that the involution of skin lesions after treatment also result in regression of angiogenesis and in particular, the regression of neovascularization. When LL lesions, which were treated more than ten years ago, were compared to residual lesions, it was observed that the inflammatory infiltrate was minimal, with a score of zero. The number of vessels (CD31+) and neovessels (CD105+) were also similar to that of the control group (Tables 1 and 3, Figure 2e and 2f). Neovessels are induced and actively maintained when the process is active, such as in LL. After treatment, granulomas become regressive and the remission of the neovascularization process is observed. In residual lesions, the skin architecture is preserved, showing rare residual granulomas and capillaries with morphology and number (CD31+ and CD105+) similar to the control group (Table 1, Figures 2e and 2f).

Monoclonal antibodies against different epitopes of the CD105/endoglin are being currently being developed, and clinical trials with these antibodies are being proposed or are in their initial test phases [25,26]. The results of the present study show that CD105+ neovessels are present across the leprosy spectrum and in the reactional forms. There is a direct relationship between the amount of CD105+ vessels and the extension of inflammatory infiltrate, as well as in reactional forms and its corresponding forms on the spectrum. These drugs could be a potential improvement in therapy, as the most commonly used drugs for the treatment of reactions, such as thalidomide and corticosteroids, are potentially teratogenic or can cause major physiological disorders, resulting in diseases such as diabetes, hypertension, cataracts, obesity and bone demineralization, among others [23].

The differences in the levels of CD105 in the serum, as detected by ELISA, were not statistically significant between the forms across the spectrum, the reactional states, the residual leprosy cases and the controls (Figure 4). This suggests that in leprosy, the expression of CD105 in the endothelial cells of neovessels, which was detected in situ by IHC, is a more localized phenomenon that occurs at the lesion site. Some studies with neoplasias also show contradictory results in respect to anti-CD105 levels in the serum by ELISA with prognostic and predictive value [13]. Unfortunately, the present results do not indicate that the detection of CD105 in human serum by ELISA can be used as a parameter for the diagnosis of reaction or even to monitor the efficacy of leprosy treatment. However, further studies are needed to establish the prognostic role of CD105 in the serum of leprosy patients because to date, a reliable serological marker to predict reactional episodes, assessment of disease activity or response to treatment is still lacking.

The lymphatic vascular system (LVS) has emerged as an important player in pathological processes, such as inflammatory diseases and tumor metastasis. However, only a few studies have focused on the role of the LVS in infectious processes, and no such studies have been conducted for leprosy.

In the present study, the results show that there is lymphangiogenesis in leprosy, but it is more significantly present in the tuberculoid side of the spectrum, especially in the polar TT form (Table 1, Figures 2g, 2h and 3). The lymphatic vessels are mainly observed in the superficial dermis and around the tuberculoid granulomas but rarely within the granulomas. Lymphangiogenesis on the tuberculoid side of leprosy spectrum may be associated with the histological profile of granulomas, which are composed of activated epithelioid macrophages, surrounded by a dense lymphocytic "B" and "T" infiltrate and low bacillary index (0 and 1+). Therefore, the lymphangiogenesis observed in the tuberculoid side of leprosy spectrum could contribute to increase the trafficking of cells and antigens, amplifying the immune response and the bacillary clearance, in a tentative to restrain the disease process. Given the scarcity of data the involvement of lymphatic vessels in inflammatory and infectious processes requires further study.

In conclusion, there is angiogenesis across the leprosy spectrum as well as in the reactional states. In situ, angiogenesis is more closely related with the extension of the inflammatory infiltrate than with forms of the spectrum or BI. There are no significant differences in the values of serum CD105 detected by ELISA. Lymphangiogenesis is present in the tuberculoid side of leprosy. The post-treatment regression of lesions is followed by the regression of neovascularization. Neovascularization is present across the spectrum of leprosy, especially on the lepromatous side and reactional states, indicating that anti-angiogenic drugs, including biological anti-CD105 may be useful in the treatment of primary leprosy cases, the prevention of reactional states and the treatment of established reactional episodes.
